# Neutrophil–lymphocyte ratio and platelet–lymphocyte ratio as potential predictive markers of treatment response in cancer patients treated with immune checkpoint inhibitors: a systematic review and meta-analysis

**DOI:** 10.3389/fonc.2023.1181248

**Published:** 2023-10-26

**Authors:** Tibera K. Rugambwa, Omar Abdihamid, Xiangyang Zhang, Yinghui Peng, Changjing Cai, Hong Shen, Shan Zeng, Wei Qiu

**Affiliations:** ^1^ Department of Oncology, Xiangya Hospital, Central South University, Changsha, Hunan, China; ^2^ Department of Internal Medicine, Mbeya Zonal Referral Hospital and Mbeya College of Health and Allied Sciences, University of Dar-es-salaam, Mbeya, Tanzania; ^3^ Garissa Cancer Center, Garissa County Referral Hospital, Garissa, Kenya; ^4^ Department of Oncology, The First People's Hospital of Loudi, Loudi, Hunan, China

**Keywords:** neutrophil-lymphocyte ratio, platelet-lymphocyte ratio, immune checkpoint inhibitors, predictive, biomarkers, response

## Abstract

**Background:**

The role of platelet–lymphocyte ratio (PLR) and neutrophil–lymphocyte ratio (NLR) as independent prognostic markers in different tumors is well established. However, there is a limited review of the potential of NLR and PLR as predictors of treatment outcomes from immune checkpoint inhibitors (ICIs).

**Objective:**

To establish a correlation between NLR and PLR and the potential of clinical benefit from ICIs.

**Methods:**

The literature search was performed for studies that reported the association between NLR, PLR, and treatment outcomes among cancer patients treated with ICIs. The outcomes of interest were objective response rate (ORR), disease control rate (DCR), and progressive disease (PD). ORR was the summation of patients who achieved complete response and partial response. DCR included patients who achieved stable disease. PD was the proportion of patients who progressed, relapsed, or discontinued the treatment. Statistical analysis was performed using the STATA 12.0 package. Heterogeneity was determined by the I^2^ value. Quality assessment was performed using the Newcastle–Ottawa Scale. Egger’s test was used to establish publication bias and sensitivity analysis.

**Results:**

A total of 40 papers that met the inclusion criteria were included in the systematic review. However, only 17 studies were used in the meta-analysis to determine the correlation between NLR, PLR, and treatment response. We found that treatment with ICIs and monitoring of outcomes and adverse events using PLR and NLR parameters have been studied in different tumors. Our analysis showed that low NLR correlated with higher ORR (OR = 0.62 (95% CI 0.47–0.81, p = 0.001) and higher DCR (OR = 0.23, 95% CI 0.14–0.36, p < 0.001). Higher NLR predicted a higher probability of PD (OR = 3.12, 95% CI 1.44, 6.77, p = 0.004). Similarly, low PLR correlated with higher ORR (OR = 0.69, 95% CI 0.5, 0.95, p = 0.025). Generally, patients with low NLR and PLR were more likely to achieve clinical benefit and better response (p-value < 0.001). Meanwhile, patients with high ratios were more likely to progress (p-value < 0.005), although there was significant heterogeneity among studies. There was no significant publication bias observed.

**Conclusion:**

The study showed that high NLR and PLR either at baseline or during treatment is associated with poorer treatment outcome. Therefore, these ratios can be utilized in clinical practice with other markers to determine treatment efficacy from immunotherapy.

## 1 Introduction

Chronic inflammation is one of the enabling characteristics in the acquisition of hallmarks of cancer, together with genomic instability (1). The inflammatory process is driven by key inflammatory cells, namely, lymphocytes, neutrophils, monocytes, and platelets (2). Interaction of these cells in the tumor microenvironment (TME) and the peripheral circulation not only facilitates the propagation and survival of cancer cells but also provides them with the ability to evade the immune system, induce angiogenesis, and metastasize to other sites (2).

Immunotherapy is one of the pillars of cancer treatment in combination with surgery, chemotherapy, radiotherapy, and the expanding targeted therapy. These drugs function by blocking immune checkpoints, which are programmed death-1 (PD-1) and its ligand (PDL-1), cytotoxic T-lymphocyte antigen 4 (CTLA-4), and lymphocyte-activation gene 3 (LAG-3), resulting in upregulation of T-cell activation, preventing tumor evasion and increasing CD8 T-cell response toward cancer cells (3).

Indication of immune checkpoint inhibitors (ICIs) is expanding rapidly from advanced disease settings to neo-adjuvant and adjuvant use in early disease (4–6) with the potential of complete treatment response and durable disease control in some patients (3). Currently, ICIs are indicated for multiple cancers with non-small cell lung cancer (NSCLC) and melanoma deriving the greatest benefit to gastrointestinal, genitourinary, and breast cancers and lymphomas just to mention a few (3).

The mechanism of action of immunotherapy depends on the inflammatory cells and tumor immunogenicity (3). Hence, in a state of lymphopenia (7), thrombocytosis (8, 9), and neutrophilia (10) either at baseline or during the course of treatment as is the case in most patients with advanced disease and poor performance status, it is less likely to achieve durable clinical response. In addition, tumors that can generate significant immune responses like melanoma and squamous cell carcinomas show dramatic responses in comparison to cold tumors like gliomas and pancreatic cancer (3).

The currently approved biomarkers to predict response to immunotherapy are PDL-1 levels, microsatellite instability status (MSI), and tumor mutation burden (TMB). However, these have been shown to be applicable in a small proportion of patients (2, 11). Although they have revolutionized the use of ICIs in cancer treatment, the fact that they are tissue-based makes them susceptible to tumor heterogeneity (12). In addition, they cannot distinguish between patients who will respond to therapy against those who will not (12).

Interaction between neutrophils, platelets, and lymphocytes reflects the balance between protumoral inflammation and anti-tumor activity (3). In some studies, the neutrophil–lymphocyte ratio (NLR) and platelet–lymphocyte ratio (PLR) was associated with a better response than PDL-1 levels (11, 13). Therefore, there is a need to develop a prognostic and predictive model that incorporates other potential biomarkers to be able to determine those who are more likely to benefit from treatment (14, 15).

A recent meta-analysis looked at the association of dynamic changes in NLR with survival outcomes and treatment response (16). The study concluded that lower baseline NLR and a downward trend of NLR during and post-treatment with immunotherapy were associated with longer survival and better tumor response (16). However, very few studies that were included reported on treatment response and disease control. Another meta-analysis on renal cell carcinoma (RCC) also showed high NLR correlated with worse survival outcomes (17).

Notably, a significant number of studies have focused on the role of NLR and PLR as prognostic factors, but very few have focused on treatment response with immunotherapy (18–21). Therefore, this study will focus on the role of NLR and PLR as predictive markers of response to immunotherapy.

## 2 Methods

### 2.1 Search strategy

The systematic review and meta-analysis were conducted in accordance with Preferred Reporting Items for Systematic Reviews and Meta-analyses (PRISMA) guidelines. A comprehensive literature search was conducted from PubMed, Embase, Web of Science, and Cochrane Library from 2015 to 2022. The search terms employed were “neutrophil-to-lymphocyte ratio” AND “immune checkpoint inhibitors” and “platelet-to-lymphocyte ratio” AND “immune checkpoint inhibitors” (**Table S1**).

The outcomes of interest were objective response rate (ORR), disease control rate (DCR), and progressive disease (PD) as defined by Response Evaluation Criteria in Solid Tumors (RECIST) 1.1. ORR was the summation of patients who achieved complete response and partial response. DCR included patients who achieved stable disease. PD was the proportion of patients who progressed, relapsed, or discontinued the treatment. The correlation was made according to cutoff values of NLR and PLR established at baseline and during the course of treatment as determined by the authors.

### 2.2 Inclusion and exclusion criteria

Studies that met the following criteria were included in the study:

□ studies published from 2015 to 2022;
□ studies that enrolled patients with solid tumors who received any of the ICIs;
□ studies reporting clinical response (ORR, DCR, and PD) and prognostic value of inflammatory markers and ICIs; and
□ prospective studies, retrospective studies, exploratory studies, and randomized controlled trials (RCTs).

#### 2.2.1 Exclusion criteria

□ Studies that did not document or analyze the association or prognostic value of inflammatory markers and ICIs;
□ non-English studies;
□ abstracts, reviews, meta-analyses, case reports, editorials, letters to the editor, and commentaries; and
□ animal studies.

### 2.3 Data extraction

The following information was extracted:

□ name of the first author,
□ year of publication,
□ type of cancer,
□ number of patients,
□ type of study design,
□ inflammatory markers investigated, and
□ numerical data for NLR, PLR, ORR, DCR, and PD from frequency tables.

### 2.4 Quality assessment

The Newcastle–Ottawa Scale (NOS) was used to assess the quality of included studies. Any study with a minimum of two stars was considered suitable to be included in the review and meta-analysis. However, the most important criterion was the availability of quality extractable data from an individual study (**Table S2**).

### 2.5 Statistical analysis

Authoritative statistical software (Stata 12.0: StataCorporation) was used to perform the meta-analysis. The OR and 95% CI values were applied to estimate the prognostic value of NLR and PLR for patients treated with ICIs. Individual OR and 95% CI values were combined to an overall OR and 95% CI. An OR < 1 indicated a better treatment outcome. The Higgins I^2^ statistic was applied to detect the heterogeneity between studies; p ≤ 0.1 and I^2^ > 50% indicated a substantial heterogeneity between studies, and random-effects models were adopted. Egger’s test and visual inspection of a funnel plot were carried out to evaluate the possibility of publication bias. Egger’s test result was the primary indicator, and a symmetry funnel plot with a p-value ≥0.05 was considered an insignificant publication bias.

## 3 Results

### 3.1 Literature screening results

**Figure 1:**
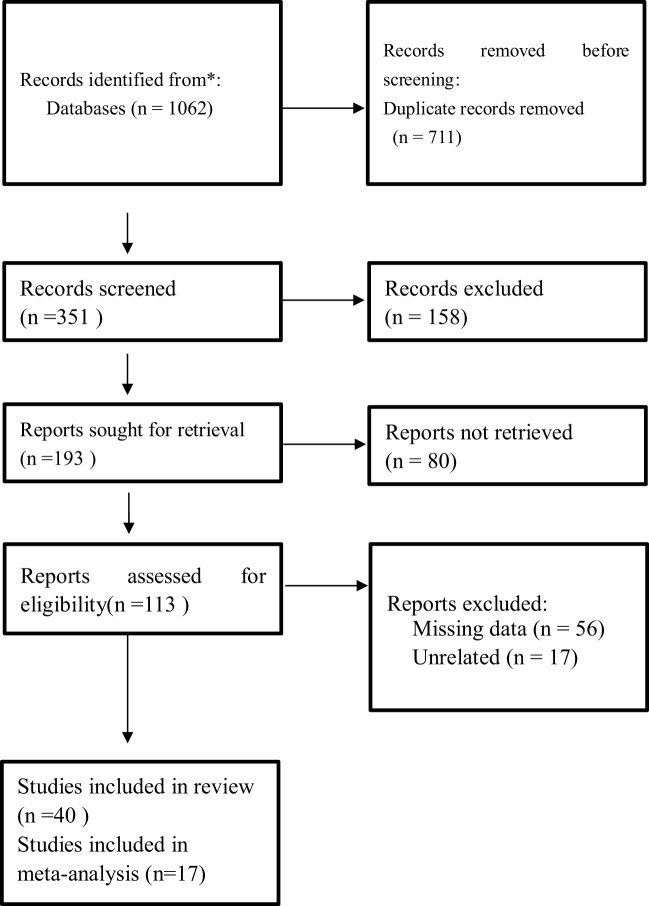
PRISMA flow diagram of study selection for inclusion in the systematic review and meta-analysis. PRISMA, Preferred Reporting Items for Systematic Reviews and Meta-analyses.

The literature search identified 1,062 studies from the database and registers. Out of those, 711 were removed as duplicates, 158 records were removed because they were not eligible, 80 reports could not be retrieved, and 73 reports either had missing information or were not related to the study. The final review and meta-analysis included 40 studies and 17 studies, respectively (**Figure 1**). The characteristics of studies, data extracted, and patient characteristics involved in the studies are represented in **Tables 1** and **2**. Important findings from the studies are summarized in **Supplementary Table 3**.

**Table 1:** Study Characteristics.

Author	Year	Design	Cancer	Patients	Marker	Outcome	NOS	Ref.no
Benzekry	2021	RC	NSCLC	298	NLR, PLR	DCR	6	20
Booka	2022	RC	GI	61	NLR, PLR	DCR,PD	8	35
Chen	2021	RC	NSCLC	151	NLR	ORR, DCR	6	15
Cheng	2022	RC	CERVICAL	70	NLR	ORR	8	54
Criscitiello	2020	RC	PAN CANCER	153	NLR, PLR	ORR, DCR	6	52
Dusselier	2019	RC	NSCLC	59	NLR, PLR	DCR-long responders PD-Early progressors	8	22
Eso	2021	RC	HCC	40	NLR	ORR, DCR, PD	8	44
Faccinetti	2018	PC	NSCLC	54	NLR	DCR, PD	6	23
Fan	2021	RC	GI	111	NLR, PLR	ORR, DCR, PD	8	36
Guida	2021	RC	MELANOMA	331	NLR	DCR	6	42
Guida	2022	RC	MELANOMA	272	NLR	DCR	6	43
Guven	2022	RC	PAN CANCER	231	NLR	ORR	8	53
Huang	2020	RC	NSCLC	61	NLR	DCR, PD	8	24
Hung	2021	RC	HCC	45	NLR, PLR	DCR, PD	8	45
Jiang	2020	RC	NSCLC	76	PLR	DCR, PD	8	11
Jung	2017	RC	MELANOMA	104	NLR	DCR, PD	8	75
Kim	2022	RC	GASTRIC	45	NLR	ORR, DCR	9	37
Lee	2021	RC	HNSCC	45	NLR	ORR, DCR	6	50
Moller	2021	RC	NSCLC	90	NLR	DCR, PD	7	26
Mountzios	2021	RC	NSCLC	672	NLR	ORR, DCR	7	27
Musaelyan	2022	RC	NSCLC	45	NLR, PLR	DCR-Responders PD-Non-responders	7	10
			MELANOMA	29				
Nakazawa	2022	RC	GASTRIC	58	NLR	DCR, PD	7	38
Namikawa	2020	RC	GASTRIC	29	NLR	DCR	7	39
Nenclares	2021	PC	HNSCC	100	NLR	DCR-Responders PD-Non-responders	6	51
Newman	2020	RC	NSCLC	137	NLR	DCR, PD	8	28
Ohashi	2020	RC	MELANOMA	16	NLR	ORR, PD	6	41
Ohba	2019	RC	NSCLC	32	NLR	ORR, DCR	8	29
Petrova	2020	RC	NSCLC	119	NLR, PLR	DCR, PD	9	31
Pu	2021	RC	NSCLC	184	NLR, PLR	ORR, DCR	8	32
Quaquarini	2022	RC	NSCLC	166	NLR	DCR, PD	9	33
Rebuzzi	2022	RC	RCC	422	NLR, PLR	ORR, DCR, PD	7	47
Russo	2018	RC	NSCLC	62	PLR	ORR	6	9
Simonaggio	2020	RC	RCC	86	NLR	DCR, PD	7	48
Spassova	2022	RC	MERKEL	114	NLR	DCR, PD	7	55
Tanaka	2022	RC	HCC	28	NLR	ORR-Responders PD-Non-responders	6	46
Wang	2022	RC	ESCC	69	NLR	ORR, DCR	8	40
Wu	2021	RC	NSCLC	136	NLR, PLR	ORR, PD	6	34
Yamamoto	2020	RC	UC	121	NLR, PLR	ORR	6	49
Yuequan	2021	RC	NSCLC	103	NLR, PLR	PR vs PD SD vs PD	8	76

RC, Retrospective cohort; PC, Prospective cohort; GI, Gastroinstestinal cancer; HCC, Hepatocellular carcinoma; HNSCC, Head and neck squamous cell carcinoma; RCC, Renal cell carcinoma; UC, Urothelial carcinoma; NLR, Neutrophil-to-lymphocyte ratio; NSCLC, Non-small cell lung cancer; CRC, Colorectal cancer; ESCC, Esophageal squamous cell carcinoma; PAN CANCER, Multiple cancers; PLR, Platelet-to-lymphocyte ratio; NOS, Newcastle Ottawa scale.

**Table 2:** Characteristics of patients involved in the studies that related NLR, PLR and treatment response.

Author, Country		Tumor type	Gender (M/F)	Age	ECOG PS High 0-1 Low >1	NLR values		PLR values	Treatment
Booka, Japan		Upper GI	49/12	<65-11 >65-50	PS<1-49 PS>2-12	3.9(0.9-31.7)		118(31-860)	Nivolumab Pembrolizumab
Benzekry, France		NSCLC	199/99	Median 62 (55,69)	Low-26 High-265	Mean-5.66 Median-3.85		Mean-273 Median-214	ICI-295 Comb-3
Chen, China		NSCLC	115/36	<63-70 >63-81	High-147 Low-4	>2.96=75 <2.96=76 (median)		>159=75 <159=76 (median)	ICI+Chemo=105 ICI+Angio=18 Both=28
Cheng, China		CERVIX	F=70	Median 51(29-77)	N/R	5.17(3.19-9.16)		270.5(174.19-363.49)	PD1+chemo=21 PDI+CHEMO+Angio=49
Christiello, Italy		-GI -Breast -Gynacologic -HNSCC -NSCLC -Melanoma& other skin cancers -MesothelICIma -NET -GUT	62/91	Median 58(31-80) >65=46 <65=107	Low=71 High=82	6		300	ICI=59 ICI+ICI=84 ICI+TARGET=10
Dusselier, France		NSCLC	44/15	Median 59.5(30.3-87.3)	Low=6 High=53	<5=21 >5=37		<160=19 169-262=31 >2=8	Nivolumab
Eso, Japan		HCC	35/5	Median 70.5(53-82)	N/R	2.56(0.39-14.0)		125(27.1-351)	Atezo/Bev
Facchinetti, Italy		NSCLC	45/9	Median 69(43-85)	LOW-15 High=39	To be retrieved		To be retrieved	Nivolumab
Fan, China		GI	56/55	>65=23 <65=88	N/R	>5=17 <5=94		<135=55 >135=56	ICI+Chemo=74 ICI+Target=44 ICI+RT=7
Guida, Italy		Melanoma	204/127	Median 63.4(53.3-73.8)	Low=78 High=252	NR		NR	Anti-PD1=246 Ipilimumab=80 Anti-PD1+Ipi=5
Guida, Italy		Melanoma (BRAF wt)	172/100	Median 63.2(52.0,73.0	Low=2 High=270	ΔNLR=0.86		ΔPLR=22.85	PD-1=209 CTLA4=57 PD-1+CTLA4=6
Guven, Turkey		Melanoma RCC NSCLC Others	155/76	Median 61(51-67)	Low=30 High=201	<5,<10% increase=76 >5, >10% increase=155			Niv=169 Atezo=28 Pembro=20 Ipi=13 Ave=1
Huang, China		NSCLC	38/23	>.65=11 <65=50	High=60 Low=1	MEDIAN C1-2.72 C2=2.93 C3=2.56 C4=2.69			Niv=24 Pembro=6 Atezo=27 Niv/ipi=4 ICI+Chemo=5
Hung, China		HCC	41/4	61.8+/-9.6	Low=1 High=44	Serum NLR 4.0+/-2.2			Nivolumab
Jiang, China		NSCLC	66/10	61(35-74) median	Low=7 High=69			>168.13=27 <168.13=41	Niv=59 Durvalumab=17
Jung, Korea		Melanoma	51/53	58(50-66) median	Low=12 High=92	<5=84 >5=20			Ipilimumab
Kim, Korea		GC	34/11	Median 60(23-76)	NR	<2.9=23			Nivolumab
Lee, Korea		HNSCC	103/22	Median Median 57(33-87)	Low=19 High=106	>4=49 <4=76			PD-1=73 PD-L1=24 PD1/PDL1+CTLA4=28
Moller, Germany		NSCLC	60/30	Median 65(31-87)	High=90 Low=0	<6.1=61 >6.1=29			pembrolizumab
MountzICIs, Greece Germany		NSCLC	463/209	65 (median)	High=584 Low=88	Median 4.8(8.1)			ICI=460 ICI+Chemo=212
Musaelyan, Russia		NSCLC Melanoma	46/28	Median 62(59-69) 57(53-62)	N/R	N/R			Niv=41 Pembro=30 Atezo=3
Nakazawa, Japan		GC	45/13	Median=66	0=8 >1=50	**Baseline** DC 3.18+/-0.65 PD 4.85+/-0.49 After C2 DC 2.97+/-0.8 PD 5.43+/-0.7			Nivolumab
Namikawa, Japan		GC	19/10	Median 71(49-86)	High=28 Low=1	Baseline 1.8(0.5-9.4) Week 8 2.5(0.9-13.2)			nivolumab
Nenclares, UK		HNSCC	80/20	Median 62(31-85)	N/R	Baseline-responders(mean) 6.4+/-6.5 Non-responders 9.1+/-10.22)			ICI not specified
Newman, USA		NSCLC	80/57	Median 68.4(28-92)	N/R	Baseline <5=90 >5=47			1^st^ line ICI=25 >2^nd^ line=112 ICI+Chemo=8
Ohashi, Japan		Melanoma	8/8	Median 74.6(51-88)	High=15 Low=1	Baseline NLR Responders-2.7(1.6-3.7) Non-responders-2.3(1.4-3.3)			Nivolumab pembrolizumab
Ohba, Japan		NSCLC	29/3	<70=26 >70=6	High=30 Low=2	Median 4.16(0.98-109.15) <4.11=19 >4.11=13			pembrolizumab
Park, Korea		NSCLC	62/21	65(42-82)	N/R	Cut-off value baseline 4.0		Cut off value baseline= 210	pembro=18 atezo=65
Petrova,Bulgaria		NSCLC	74/45	62.3+/-7.9	High=119 Low=0	Median NLR <5=57 >5=62		MEDIAN PLR <200=60 >200=59	Pembro
Pu China		NSCLC	134/50	Median 58(33-87) <70=153 >70=31	High=174 Low=10	NLR<5=115 >5=69		<200=99 >200=85	Pembro=98 Niv=86
Quaquarini, Italy		NSCLC	129/37	<65=54 >65=54	High=147 Low=19	<5=81 >5=85			Niv=84 Pembro=56 Atezo=26
Rebuzzi, Italy		RCC	305/117	Median 63.4(18-85) <70=314 >70=108	KPS>80%=367 KPS<80%=55	Mean=4.12		Mean=237	nivolumab
Russo, Italy		NSCLC	24/4	69(47-78)				PLR>160=2 PLR<160=12	nivolumab
Simonaggio, France		MRCC	67/19	Median 67(21.6-82)	High=73 Low=12	Median(95%CI) 3.26(1-37)			Nivolumab
		MNSCLC	47/28	65(31.2-86.7)	High=51 Low=24	Median 3.4(1.4-13)			Nivolumab
Spassova, Germany		MERKEL CELL CARCINOMA (MCC)	82/32	<70=40 >70=74	PS-0=64 PS>1=49 Not available=1	<4=54 >35=35 Not available=25			Avelumab=57 Niv=13 Pembro=44
Tanaka, Japan		HCC	22/6	73.5(56,89)	High=27 Low=1	3.13(1.19-23.7)			Atezo/Bev
Wang, China		ESCC	64/5	61(38-75)	PS-0=47 PS-1=22	NLR<4=36 NLR>4=33			Camrelizumab
Wu, China		NSCLC	101/35	<60=75 >60=61	HIGH PS<1=124 LOW PS>2=12	REPORTED IN TERMS OF DELTA (pre,medICI, post)			ICI-not specified Absolute values not provided
Yamamoto, Japan		UC	87/34	74(50-86)	Not provided	NLR cut off=3	PLR cut off=154		Pembro
Yuequan, China		NSCLC	68/35	Median 66(61,71)	High=97 Low=6	<5=69 >5=34			ICI=32 ICI+Chemo=71

Ave, Avelumab; Atezo, Atezolizumab; Bev, Bevacizumab; Chemo, chemotherapy; Comb, combination; ECOG-PS, Eastern Cooperative Oncology Group; GC, Gastric cancer; GI, Gastroinstestinal, GUT, Genitourinary tract; HCC, Hepatocellular carcinoma; HNSCC, Head and neck squamous cell carcinoma; ICI, Immune checkpoint inhibitor; Ipi, Ipilimumab; KS, Karnofsky status; M/F, Male/female; NET, Neuroendocrine tumor; NLR, Neutrophil-lymphocyte ratio; Niv, nivolumab; NSCLC, non-small cell lung cancer; N/R, not recorded; PD-1, Programmed death-1; PD-L1, Programmed death ligand 1; PLR, Platelet-lymphocyte ratio; Pembro, pembrolizumab; RCC, renal cell carcinoma; WT, wild type.

### 3.2 Research characteristics

A total of 40 studies were included in this systematic review, but only 17 studies (13, 22–37) qualified for meta-analysis. Regionwise, almost half of the included studies came from Asian countries (Japan, China, and Korea). The sample size in the included 40 studies ranged from 16 patients to 672 patients. Almost all studies were retrospective in nature except for one study that used a prospective study design (23) and received moderately high scores in the Newcastle–Ottawa Scale quality assessment. A total of 36 studies looked at distinct cancer types, and three studies looked at two or more types of tumors. Out of the 36 studies that looked at specific cancer types, NSCLC was the most frequently studied tumor. Fifteen studies focused only on ICI as a single agent, while the remaining studies included patients who received immunotherapy in combination with other cancer treatment modalities. Apart from reporting NLR and PLR, other blood cell counts included derived neutrophil–lymphocyte ratio (dNLR), absolute lymphocyte count (ALC), absolute neutrophil count (ANC), absolute platelet count (APC), absolute eosinophil count (AEC), and leukocyte count and its differentials. Receiver operating characteristic (ROC) curves were used in nine studies (25, 33, 36, 38–43) to determine the optimal cutoff value for NLR and PLR.

#### 3.2.1 Non-small cell lung cancer

Up to 50% (18 studies) of the included studies (10, 11, 13, 22–28, 38, 39, 44–49) reported the association of NLR and PLR in non-small cell lung cancer patients treated with ICIs. The sample size ranged from 45 to 672 patients. The average number of patients was 142 patients. In total, there were 2,563 patients. One study only reported the ORR, five studies reported on DCR, four studies reported on ORR and DCR, and seven studies reported DCR and PD. None of the included studies had all the three components. Two studies compared numerical percentages between patients who responded to treatment (DCR) and those who progressed (PD). One study reported the correlation of ratios with overall short-term efficacy.

The studies were further divided on the basis of the presence or absence of a significant relationship between the ratios and end points. The subdivision produced a total of 51 reports. Out of those, five reports were on ORR, 27 reports were on DCR, and 19 reports were on PD.

##### 3.2.1.1 Objective response rate

Three out of five reports showed a statistically significant relationship between the ratios and ORR. In one of the studies, ORR was higher in PLR-low patients compared to PLR-high patients (46.15% *vs.* 8.3%, p < 0.0004). In another study, those who had a decrease in NLR 12 weeks post-treatment were more likely to derive clinical benefit than those with increasing NLR (OR = 3.304, 95% CI 1.560–7.001, p = 0.002).

##### 3.2.1.2 Disease control rate

Out of 29 reports, 16 reports showed a significant correlation between NLR, PLR, and DCR, while nine studies reported a lack of relationship. Most of the reports with significant association also noted a higher DCR among patients with low ratios either at baseline or after a few cycles of treatment.

##### 3.2.1.3 Progressive disease

Ten reports showed a statistically significant correlation between the ratios and PD. Generally, patients with higher NLR and PLR tend to progress earlier and have a higher rate of PD as compared to those with low ratios.

#### 3.2.2 Gastrointestinal cancer

Six studies (29–32, 50, 51) (17 reports) reported the correlation between ratios and study end points. Out of five reports for ORR, only one study showed a significant relationship with NLR at baseline compared with other times (NLR L *vs.* H Baseline 36.1% *vs.* 9.1%, p = 0.018; at V1 34.4% *vs.* 15.6%, p = 0.083; variation (baseline-V1) < 20% *vs.* >20%; 31.6% *vs.* 22.2%, p = 0.430).

Among nine reports that looked at DCR, five of them showed a positive correlation, while four reports lacked a statistically significant relationship. Those with low PLR and NLR had higher DCR than those with high ratios (PLR L *vs.* H) = 36.7% *vs.* 9.7%, p = 0.012; NLR (L *vs.* H) = 33.3% *vs.* 12.9%, p = 0.058).

Likewise, patients with higher ratios had higher rates of progressive disease than those with low ratios. In a retrospective study of 58 patients (50), mean NLR was significantly higher in the PD group at both baseline and post-treatment (Pre-rx, 318 *vs.* 4.85, p = 0.045; Post-rx, 2.97 *vs.* 5.43, p = 0.025). In another study (51), NLR showed a statistically significant relationship at week 4 post-treatment compared to other times (p = 0.044).

#### 3.2.3 Melanoma

A study by Ohashi et al. with a small sample size of 16 patients reported no significant relation between NLR and ORR (52).

Another retrospective study (53) that looked at DCR reported a significant correlation between NLR and DCR despite the NRAS mutation status (OR = 0.88, 95% CI 0.77–1.00, p = 0.005). Also, a change in NLR and PLR correlated with lower response (ΔNLR with OR = 2.779, p < 0.001, ΔPLR OR = 2.022, p < 0.009) (40).

#### 3.2.4 Hepatocellular carcinoma

Out of the three studies (33, 41, 54) (10 reports), three reports on DCR and three reports on PD showed a significant relationship. Only one study reported a lack of correlation between the ratios and ORR.

#### 3.2.5 Urothelial carcinoma (renal [RCC] and bladder [UC])

Rebuzzi et al. (34) reported mean values of NLR and PLR among patients who achieved clinical response and those with progressive disease at baseline and after four doses of treatment (longitudinal variation). The mean value of NLR and PLR at baseline and after four doses of treatment was lower as compared with the group with progressive disease (NLR, 3.18 *vs.* 4.12, p = 0.012; PLR, 184 *vs.* 237, p = 0.003).

In the study by Simonaggio et al. (55), the NLR-low group had greater DCR in any NLR decrease at week 6 as compared to the NLR-high group (81% *vs.* 40%, p = 0.0007).

Likewise, in the study by Yamamoto et al. (42), NLR had a statistically significant association with ORR (p = 0.016), while PLR had a marginal significance (p = 0.0536).

#### 3.2.6 Head and neck cancers

A study by Lee et al. (56) showed that those patients with high NLR were associated with poor response (OR = 0.3, 95% CI 0.11–0.84, p = 0.022). Similar findings were reported by Nenclares et al. (43) where NLR was significantly lower in responders (DCR) compared to non-responders (p < 0.001).

#### 3.2.7 Across solid tumors

In both pan-solid cancer retrospective studies (35, 57), those with low ratios had a greater response rate as compared to those with high ratios. In a study by Guven et al., those patients with high NLR and greater than 10% NLR increase had the lowest ORR.

#### 3.2.8 Other tumors

One study on cervical cancer (36) reported a significant relationship between NLR and ORR [NLR (L *vs.* H) 78.26% *vs.* 53.19%, OR = 0.316, 95% CI 0.1–0.991, p = 0.048], while there was no relationship with PLR [PLR (L *vs.* H) 70% *vs.* 58%, OR = 0.592, 95% CI 0.195–1.794, p = 0.354]. However, despite the lack of significance, ORR was higher in patients with low ratios than those with high ratios.

A study on advanced Merkel cell carcinoma by Spassova et al. (37) noted that the NLR-low group had more patients with disease control (ORR) than the NLR-high group (49% *vs.* 37%), while there was no difference in the group with disease progression.

### 3.3 Meta-analysis

**Figure 2:**
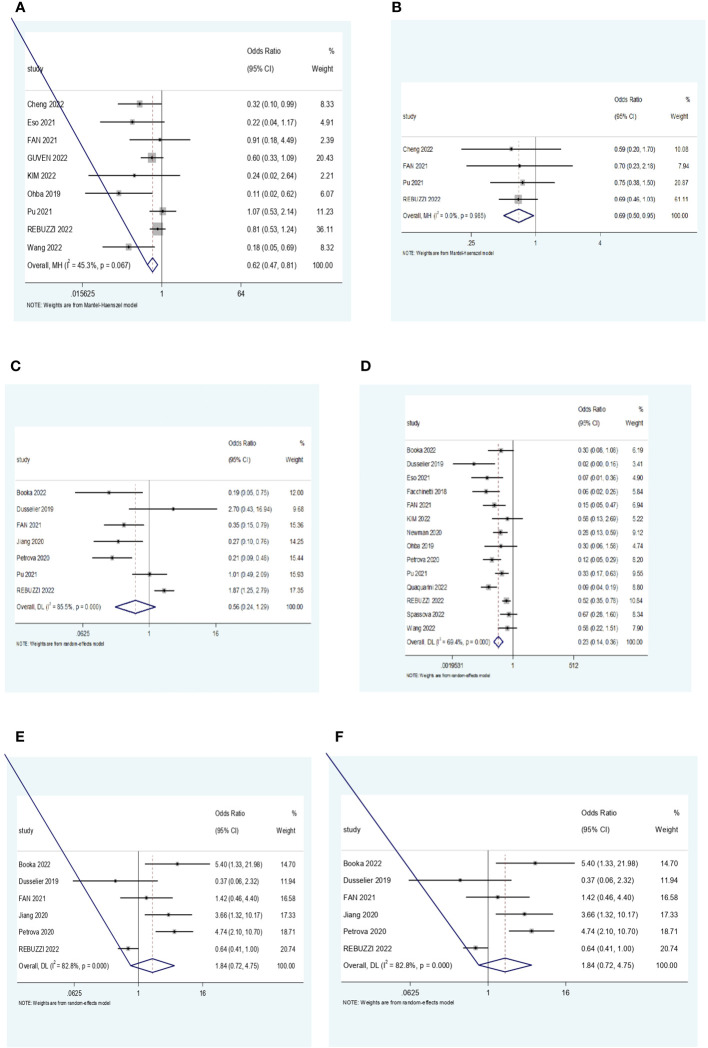
**(A)** Forest plot for the association NLR and ORR. **(B)** Forest plot for the association between PLR and ORR. **(C)** Forest plot for the association between NLR and DCR. **(D)** Forest plot for the association between PLR and DCR. **(E)** Forest plot for the association between NLR and PD. **(F)** Forest plot for the association between PLR and PD. NLR, neutrophil–lymphocyte ratio; ORR, objective response rate; PLR, platelet–lymphocyte ratio; DCR, disease control rate; PD, progressive disease.

As described in the Methods, a meta-analysis was conducted under two subgroups NLR and PLR for the treatment efficacy end-point ORR, DCR, and PD. Each study that focused on these inflammatory markers was assessed independently. Forest plots were used to represent the pooled results (**Figures 2A–F**).

#### 3.3.1 Meta-analysis for ORR, DCR, and PD in NLR subgroup

A total of nine studies looked at the impact of NLR on ORR. All studies showed a positive correlation between a low NLR and a higher ORR. Out of the nine studies, five of them showed statistical significance (p < 0.05). The pooled effect estimate (OR) was found to be statistically significant at a value of 0.62 (95% CI 0.47–0.81, p = 0.001). Assessment of heterogeneity suggests that there is low heterogeneity between the studies included in the subgroup meta-analysis (I^2^ = 45.3%, p = 0.067).

Analysis of DCR included 14 studies, whereby 10 of them showed a statistically significant correlation in favor of low NLR, suggesting that patients with low ratios were more likely to have a better treatment response. The pooled effect estimate (OR) was found to be statistically significant at a value of 0.23 (95% CI 0.14–0.36, p < 0.001). Assessment of heterogeneity suggests that there is high heterogeneity between the studies included in the subgroup meta-analysis (I^2^ = 69.4%, p = 0.000).

Ten studies reported the correlation of NLR with progressive disease. Eight studies demonstrated that higher NLR was associated with a higher probability of disease progression. One study had contrasting results whereby low NLR was associated with PD. The overall estimate was statistically significant at a value of 3.12 (95% CI 1.44, 6.77, p = 0.004). Assessment of heterogeneity showed substantial heterogeneity among the included studies (I^2^ = 84.8%, p = 0.000).

#### 3.3.2 Meta-analysis of ORR, DCR, and PD in PLR subgroup

Four studies were pooled to determine the impact of PLR levels on the ORR. All four studies showed a positive correlation between low PLR levels and ORR, but only one study was statistically significant. The pooled effect (OR) was found to be statistically significant at a value of 0.69 (95% CI 0.5, 0.95, p = 0.025). There was no heterogeneity among the studies (I^2^ = 0.0%, p = 0.985).

In the analysis of DCR, four studies showed a statistically significant positive correlation between low PLR and DCR, suggesting a better treatment response in PLR-low patients. Three studies favored high PLR, but only one of them was statistically significant. The overall estimate (OR) was 0.56 (95% CI 0.24, 1.29, p = 0.172), although it was not statistically significant. Assessment of heterogeneity showed high heterogeneity between the included studies (I^2^ = 85.5%, p = 0.000).

Six studies were analyzed for the relationship between PLR levels and PD. Four studies reported a positive correlation between high PLR and a higher probability of progressive disease. Meanwhile, two studies were contradicting, suggesting that low PLR levels were associated with the likelihood of disease progression. Out of the six studies, only two studies did not show a statistically significant correlation. The overall estimate was not statistically significant at a value of 1.84 (95% CI 0.72, 4.75, p = 0.205). There was substantial heterogeneity among the studies (I^2^ = 82.8%, p = 0.000).

#### 3.3.3 Publication bias

**Figure 3:**
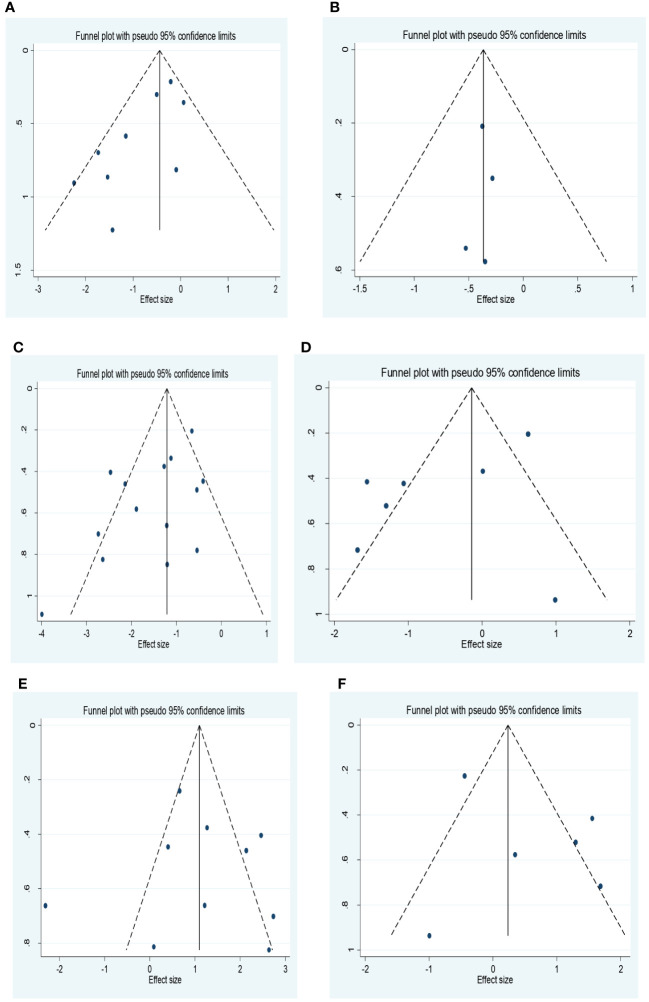
**(A)** Funnel plot for the association between NLR and ORR. **(B)** Funnel plot for the association between PLR and ORR. **(C)** Funnel plot for the association between NLR and DCR. **(D)** Funnel plot for the association between PLR and DCR. **(E)** Funnel plot for the association between NLR and PD. **(F)** Forest plot for the association between PLR and PD. NLR, neutrophil–lymphocyte ratio; ORR, objective response rate; PLR, platelet–lymphocyte ratio; DCR, disease control rate; PD, progressive disease.

Egger’s test and funnel plots were used to assess publication bias (**Figures 3A–F**). All funnel plots had a symmetrical distribution of studies. However the findings from Egger’s test showed that there was publication bias for studies that reported the association between ORR, DCR and NLR. After performing the trim and fill method, there was no significant change in the results.

## 4 Discussion

Our review and meta-analysis looked at the correlation between NLR and PLR and treatment response in patients treated with ICIs across different tumors. Generally, patients who had low ratios at baseline or decreasing trend during the course of treatment according to cutoff values pre-determined by authors based on previous studies or derived from the area under the curve (AUC) had a better treatment outcome and were more likely to obtain clinical benefit than those with higher values. Also, they had a lower rate of disease progression compared to the high-ratio group.

The findings of this study correspond with previous studies that looked at the correlation between inflammatory markers and treatment efficacy. A meta-analysis by Guo et al. studying the dynamics of NLR during ICI treatment also showed that patients with a significant upward trend of NLR did not respond to immunotherapy, while those with a downward trend were associated with better clinical and treatment outcomes (16). Similarly, Zhang et al. observed a significant correlation between NLR and ORR (p = 0.003) and a lack of significance between NLR and DCR (p = 0.111) in a meta-analysis involving patients with gastric cancer treated with immunotherapy (58).

There are limited studies on the effect of PLR and response to immunotherapy compared to NLR. Most of the literature focuses on PLR as a prognostic indicator (33, 34, 40, 41, 52–54). One of the studies included in this meta-analysis investigated blood markers before treatment that could be used as predictors of best clinical response (13). With the use of chi-square analysis, the PLR-H (<168.13) group had an inferior stable disease/partial response (SD/PR) rate than the PLR-L (<168.13). However, there were no significant differences in the best clinical response between PD-L1-positive and PD-L1-negative patients. Therefore, the study concluded that PLR could be a better predictive marker to differentiate the best response of ICIs than PD-L1 expression. Likewise in the study by Spassova et al. (37) and Musaelyan et al. (11), there was a lack of a statistically significant relationship between PDL-1 levels and clinical response. A study by Diem et al. also showed that elevated pre-treatment NLR and PLR were independently associated with poorer survival and lower response rates in lung cancer patients treated with nivolumab.

It is well-established that NLR is an independent prognostic factor in different cancers (59). The mechanism behind this observation is that some cancers express chemokines that drive the proliferation of tumor cells. Also, these chemokines drive the influx of myeloid-derived suppressor cells (MDSCs). Examples of those chemokines include CXCL5 and CXCL8, which interact with receptor CXCR2 and CXCR1 expressed on neutrophils. This influx inhibits the tumor-suppressor activity of tumor-infiltrating lymphocytes (TILs) and cytotoxic CD8+ T cells. Additionally, they promote angiogenesis and metastatic potential of cancer cells (59). A study by Kargl et al. in NSCLC patients treated with immunotherapy demonstrated that cells of myeloid origin contributed to treatment failure (60).

Tumor-associated neutrophils (TANs) present in the TME, and neutrophils present in the blood or the bone marrow are linked with resistance to immunotherapy through adaptive immune cell polarization and suppression, tumor neoangiogenesis, immune evasion and exclusion, and tumor intrinsic characteristics. TAN-rich tumors display lower macrophage and TIL infiltration, making them resistant to ICIs. In gastric cancer patients, a sub-population of neutrophils was identified in the peripheral circulation that suppresses CD8+ cell activity. Arginase-1 (ARG1)-expressing human granulocytic cells downregulate T-cell proliferation and cytokine secretion. ARG1+ neutrophils increase with tumor stage in treatment-naive patients and negatively correlate with the number of CD8+ cytotoxic T-cell lymphocytes (61).

Platelet activation is stimulated by pro-inflammatory cytokines and participates in the recruitment of neutrophils (62). They play a fundamental role in systemic and local responses against cancer. They sequester tumor molecules, including RNA and protein transcripts, altering their RNA profiles. After their interaction with the TME, they are called tumor-educated platelets. They transport material from the TME to sites closer to the tumor, creating a favorable environment for the development of metastases. They contain a rich repertoire of RNA varieties, providing biomolecules for diagnosis and prognostic, predictive, or follow-up biomarkers (62).

The prognostic and predictive roles of NLR and PLR cut across most cancer types and in all forms of cancer treatment, not only in immunotherapy. However, the lack of standard cutoff values makes them difficult to apply in clinical practice. Also, baseline values are affected by underlying pre-clinical state, co-morbid systemic conditions, and other confounders.

The findings of this study have shown how heterogeneous the utilization of NLR and PLR as prognostic and predictive factors is. The study has shown that these ratios are predictive but not in all cancers. For example, the study by Wu et al. reported a lack of correlation between inflammatory markers and immune response (63). Moreover, in the same cancer type, one factor could be predictive while the other is not, which indicates that these markers cannot be used as a single entity; rather, they are more functional when combined with other markers in a predictive or prognostic model (64).

Examples of existing models and indexes that are multivariable include neutrophil–platelet score (NPS) (65), which is a systemic inflammation score based on the number of neutrophils and platelets. When tested in NSCLC patients, NPS predicted OS and DCR in pre-treated advanced NSCLC patients who received treatment with nivolumab or pembrolizumab (65). A study by Zhao et al. showed three models, namely, lung immune prognostic index (LIPI) based on pre-treatment blood levels of derived-NLR and lactate dehydrogenase (LDH), EPSILoN (ECOG-PS, smoking, liver metastases, LDH, and NLR), and modified LIPI were predictive and prognostic in immunotherapy (64).

Another study in melanoma patients built a multivariable predictive model for response and survival. A combination of performance status, number of liver and lung metastatic sites, serum LDH, blood NLR, type of treatment (monotherapy *vs.* combination), and line of treatment was predictive of ORR (14). Another is the Gustave Roussy Immune Score (GRIm-S), which is a composite of neutrophil–lymphocyte ratio (>6 = 1), albumin (<35 = 1), and LDH (>ULN = 1) established as a prognostic score and may aid in the selection of patients for phase 1 trials of immune checkpoint inhibitors (66). Additionally, the Pan-immune inflammation value (PIV), also called the aggregate index of systemic inflammation (AISI), which combines neutrophils, monocytes, platelets, and lymphocytes, is another useful prognostic index (67, 68).

Other prognostic models and indexes utilized in overall cancer treatment include systemic immune-inflammatory index (SII), which combines platelets and NLR (20); advanced lung cancer inflammatory index (ALI), which combines body mass index and the ratio of albumin to NLR (22, 69); and the immune metabolic prognostic index, which is an association of NLR, dNLR, lymphocyte–monocyte ratio (LMR), PLR, and SII (70, 71). In genitourinary tumors, there is a FAN score in urothelial carcinoma that relates to Fibrosis-4-index, albumin–bilirubin ratio, and NLR (72). The International Metastatic RCC Database Consortium (IMDC) predictive score combines hemoglobin levels, serum calcium levels, Karnofsky performance status, time to treatment, and number of platelets and neutrophils (73). The risk blood biomarker (RBB) accounts for the total leukocyte count and ratio of neutrophils, monocytes, and lymphocytes (74). More so, the Glasgow prognostic score (GPS-m) relates to C-reactive protein (CRP), albumin, and NLR (42, 75).

Prospective studies on inflammatory cells that constitute the TME and affect treatment response continue to report other cellular markers apart from neutrophils and platelets. One study reports that more infiltration of cytotoxic CD8+ TCLs present in the intratumoral area was associated with better disease control (37). The study by Musaelyan et al. suggested that other markers of T-cell activation like IL-18 and β2-microglobulin could be used to evaluate and monitor treatment response (11). Another study used artificial intelligence-powered analysis of TILs to generate immunophenotypes, which was shown to correlate with treatment response (76). A combined model of FOXP3+ TCLs and other clinical covariates including NLR was a better predictor of response to immunotherapy in urothelial carcinoma patients (77).

Apart from inflammatory cells, the use of gene expression like circulating tumor DNA (ctDNA) kinetics (78), single-nucleotide variants (SNVs) of PD-1 and PDL-1 (79), gene expression signatures (80), and tumor burden determined from FDG-PET derived metabolic tumor volume (MTV) (81) provide additional biomarkers that predict benefit from ICIs. Pioneer trial (NCT 03493581), which is a comprehensive biomarker analysis for treatment efficacy of ICI with chemotherapy in NSCLC patients, has identified up to 15 biomarker signatures associated with efficacy and progression-free survival (PFS) (82).

This study aimed to highlight the association between inflammatory markers NLR and PLR with disease control, objective response, and disease progression for patients treated with immunotherapy. The study has highlighted that at any point in time before, during, or after treatment, both low and high ratios of NLR and PLR correlate with treatment outcomes regardless of cutoff points, something that was not reported in previous meta-analyses.

Despite the highlighted correlation, the findings are limited by the fact that almost all the included studies were retrospective in nature with a risk of information bias and publication bias. The grouping of patients according to treatment response was not homogeneous. Some studies in the systematic review were not included in the meta-analysis due to the heterogeneous nature of reported data, particularly patients with SD and those with PD. Patients with stable disease were counted with those who progressed as non-responders, while in other studies, they were counted as part of disease control. Also, there was variation in reporting of the ratios, as some studies reported the means and medians, while others just the numerical data or percentages.

Our study was heavily skewed toward NSCLC and melanoma patients, which is attributed to the fact that these were the first tumor sites to obtain Food and Drug Administration (FDA) approval to use ICIs in comparison to other sites. In addition, most patients involved in the studies were treated with pembrolizumab (anti-PD1), nivolumab (anti-PD1) and ipilimumab (anti-CTLA4), and atezolizumab (anti-PDL-1) with limited studies in other agents, hence making it challenging to generalize our findings.

In addition, most of the included studies did not report on the association between NLR and PLR and treatment response in patients treated with immunotherapy according to racial background. Therefore, determining the correlation according to racial background was not possible.

There is a paucity of literature that reported the association of NLR, PLR, and racial background in cancer patients treated with immune checkpoint inhibitors, while some studies that performed sub-group analysis according to country of origin reported contradicting results (58, 83, 84). However, sub-group analysis was not performed in this particular systematic review.

Despite the contradiction, it is evident that with effective treatment, a drop in NLR and PLR correlates with better treatment outcomes and improved survival.

## 5 Conclusion and recommendations

It is clear that the state of inflammation plays a significant role in treatment response to cancer treatment overall. Inflammatory cells serve as adjunct markers to the FDA-approved biomarkers. The fact that in some studies there was a lack of correlation between PDL-1 levels and treatment response calls for additional markers to augment the predictive and prognostic roles of PDL-1 levels, MSI status, and TMB.

These markers tend to be affected by other underlying co-morbid conditions and the overall state of the body, which compromises their prognostic and predictive functions. Therefore, there is a need to develop a comprehensive clinical model that is reflective of real-world settings and the models to be tested in clinical trials for validation before being incorporated into clinical practice.

## Data availability statement

The original contributions presented in the study are included in the article/**Supplementary Material**. Further inquiries can be directed to the corresponding author.

## Author contributions

(I) Conception and design: TR, HS, SZ, and WQ. (II) Collection and assembly of data: XZ, YP, and CC. (III) Data analysis and interpretation: OA and CC. (IV) Manuscript writing: all authors. (V) Final approval of manuscript: all authors.
