# Supplementary material for: Neutrophil–lymphocyte ratio and platelet–lymphocyte ratio as potential predictive markers of treatment response in cancer patients treated with immune checkpoint inhibitors: a systematic review and meta-analysis
Source: Front Oncol. 2023 Oct 26;13:1181248. doi: 10.3389/fonc.2023.1181248 (PMC10646751; doi:10.3389/fonc.2023.1181248)
Supplement: Supplementary file 1 [file Table_1.docx]

**Supplementary material 1; Search strategy**

| Pubmed | 1. "neutrophil-to-lymphocyte"[All Fields] AND ("ratio"[All Fields] OR "ratio s"[All Fields] OR "ratioes"[All Fields] OR "ratios"[All Fields]) AND ("immune checkpoint inhibitors"[Pharmacological Action] OR "immune checkpoint inhibitors"[MeSH Terms] OR ("immune"[All Fields] AND "checkpoint"[All Fields] AND "inhibitors"[All Fields]) OR "immune checkpoint inhibitors"[All Fields]) |
| --- | --- |
|  | 2. "platelet-to-lymphocyte"[All Fields] AND ("ratio"[All Fields] OR "ratio s"[All Fields] OR "ratioes"[All Fields] OR "ratios"[All Fields]) AND ("immune checkpoint inhibitors"[Pharmacological Action] OR "immune checkpoint inhibitors"[MeSH Terms] OR ("immune"[All Fields] AND "checkpoint"[All Fields] AND "inhibitors"[All Fields]) OR "immune checkpoint inhibitors"[All Fields]) |
| Web of Science | neutrophil-to-lymphocyte ratio (Topic) and immune checkpoint inhibitors (Topic)  platelet-to-lymphocyte ratio (Topic) and immune checkpoint inhibitors (Topic) |
| Embase | 1. ('neutrophil lymphocyte ratio'/exp OR 'neutrophil lymphocyte ratio') AND ('immune checkpoint inhibitor'/exp OR 'immune checkpoint inhibitor')  2. ('platelet lymphocyte ratio'/exp OR 'platelet lymphocyte ratio') AND ('immune checkpoint inhibitor'/exp OR 'immune checkpoint inhibitor')  Papers-72 |
