# Supplementary material for: Neutrophil–lymphocyte ratio and platelet–lymphocyte ratio as potential predictive markers of treatment response in cancer patients treated with immune checkpoint inhibitors: a systematic review and meta-analysis
Source: Front Oncol. 2023 Oct 26;13:1181248. doi: 10.3389/fonc.2023.1181248 (PMC10646751; doi:10.3389/fonc.2023.1181248)
Supplement: Supplementary file 2 [file Table_2.docx]

**Table S2; Newcastle-Ottawa Scale for quality assessment of the included studies**

| **Study** | **Selection** | **Comparability** | **Outcome** | **Total** |
| --- | --- | --- | --- | --- |
| \| \| Benzekry et al \| \| --- \| \| Booka et al \| \| Chen et al \| \| Cheng et al \| \| Criscitiello et al \| \| Dusselier et al \| \| Facchinetti et al \| \| Fan et al \| \| Guida et al \| \| Guida et al \| \| Guven et al \|   Huang et al \| \| --- \| --- \| --- \| --- \| --- \| --- \| --- \| --- \| --- \| --- \| --- \| --- \| \| Hung et al \| \| Jiang et al \| \| Jung et al \| \| Khunger et al \| \| Kim et al \| \| Lee et al \| \| Moller et al \| \| Mountzios et al \| \| Musaelyan et al \| \| Nakazawa et al \| \| Namikawa et al \| \| Nenclares et al \| \| Newman et al \| \| Ohashi et al \| \| Ohba et al \| \| Park et al \| \| Petrova et al \| \| Pu et al \| \| Quaquarini et al \| \| Rebuzzi et al \| \| Russo et al \| \| Simonaggio et al \| \| Spassova et al \| \| Tanaka et al \| \| Wang et al \| \| Wu et al \| \| Yamamoto et al \| \| Yuequan et al \| | ***  ****  ***  ****  ***  ***  ***  ****  **  ***  ****  ***  ****  ***  ***  ***  ****  ***  ***  ****  ***  ***  ***  ***  ****  ***  ***  ***  ****  ***  ***  ***  **  ***  ***  ***  ***  ***  ***  ***  ** | *  **  **  **  *  **  **  **  *  *  **  **  **  **  *  *  **  *  **  *  **  **  **  *  **  *  **  **  **  **  **  **  **  **  **  **  *  **  *  *  * | *  **  ***  ***  **  **  ***  **  **  **  **  **  **  ***  ***  **  ***  **  **  **  **  **  **  **  **  **  ***  **  **  ***  ***  **  **  **  **  **  **  ***  **  **  ** | 6  8  8  8  6  7  8  8  5  6  8  8  8  8  7  6  9  6  7  7  7  7  7  6  8  6  8  7  9  8  8  7  6  7  7  7  6  8  6  6  5 |
