# Supplementary material for: Neutrophil–lymphocyte ratio and platelet–lymphocyte ratio as potential predictive markers of treatment response in cancer patients treated with immune checkpoint inhibitors: a systematic review and meta-analysis
Source: Front Oncol. 2023 Oct 26;13:1181248. doi: 10.3389/fonc.2023.1181248 (PMC10646751; doi:10.3389/fonc.2023.1181248)
Supplement: Supplementary file 3 [file Table_3.doc]

Supplementary Table 3; Summary of studies relating NLR and PLR with treatment response

| **Author** | **Year** | **Design** | **Cancer** | **Patient** | **ORR** | **DCR** | **PD** | **Comments** |
| --- | --- | --- | --- | --- | --- | --- | --- | --- |
| Benzekry | 2021 | RC | NSCLC | 298 |  | NLR(OR=0.49,95%CI0.31,0.73)  P<0.001  PLR (OR=0.84 95%CI 0.64,1.1)  P=0.156 |  | Univariate analysis |
| Booka | 2022 | RC | UGI | 61 |  | PLR (L vs H)=36.7%vs9.7%  P=0.012  NLR (L vs H)=33.3%vs12.9%  P=0.058 | NLR (L vs H)  66.7% vs 87.1%  PLR (L vs H)  63.3% vs 90.3% |  |
| Chen | 2021 | RC | NSCLC | 151 | NLR_wk12_Down  (OR=3.304,95%CI1.560-7.001, P=0.002 | NLR_wk12_Down  OR=4.682,95%CI 0.962-22.796  P=0.056 |  | Multivariate analysis |
| Cheng | 2022 | RC | CERVIX | 70 | NLR (L vs H)  78.26%vs53.19%  (OR=0.316,95%CI 0.1-0.991)  P=0.048  PLR (L vs H)  70%vs58%  OR=0.592,95%CI0.195-1.794  P=0.354 |  |  |  |

Supplementary Table 3; Summary of studies relating NLR and PLR with treatment response(continuous)

| **Author** | **Year** | **Design** | **Cancer** | **Patient** | **ORR** | **DCR** | **PD** | **Comments** |
| --- | --- | --- | --- | --- | --- | --- | --- | --- |
| Criscitiello | 2020 | RC | PAN  CANCER | 153 | NLR (L vs H)  13.6% vs 3.7%  OR=0.24 95%CI 0.01-1.28  P=0.18  PLR (L vs H)  12.2% vs 10.8% p=0.823 | NLR (L vs H)  33.6% vs 40.7% p=0.481  PLR (L vs H)  32.2% vs 43.2% p=0.221 |  | Univariate analysis |
| Dusselier | 2019 | RC | NSCLC | 59 |  |  | Early progressors vs long responders  OR=18.08 95%CI 2.96-246.24  P=0.0014  **Baseline**  NLR (L vs H) P=0.5  PLR (L vs H) p=0.3  **Before 4^th^ infusion**  NLR (L vs H) p=0.005  PLR (L vs H) p=0.37  **Evolution**  ΔNLR>1 p=0.0007  ΔPLR p=0.008 |  |

Supplementary Table 3; Summary of studies relating NLR and PLR with treatment response(continuous)

| **Author** | **Year** | **Design** | **Cancer** | **Patient** | **ORR** | **DCR** | **PD** | **Comments** |
| --- | --- | --- | --- | --- | --- | --- | --- | --- |
| Eso | 2021 | RC | HCC | 40 |  |  | NLR (DCR vs PD)  2.47 vs 4.48  P=0.013  NLR-H pts vs NLR-L pts  NLR (H vs L-means)=4.97 vs 2.00 P<0.0001  PLR (H vs L-means)=193 vs 113  P=0.002 |  |
| Facchinetti | 2018 | RC | NSCLC | 54 |  | NLR (L vs H)  70% vs 13% | NLR (L vs H)  30% vs 87% | P<0.001 |
| Fan | 2021 | RC | GI | 111 | NLR (L vs H) p=0.909  PLR (L vs H) p=0.543 | NLR (L vs H) p=0.001  PLR (L vs H) p=0.011 |  |  |
| Guida | 2021 | RC | MELANOMA | 331 |  | NLR significant regardless of  NRAS mutation status  OR=0.88, 95%CI 0.77-1.00,  P=0.005 |  | Multivariate analysis |

Supplementary Table 3; Summary of studies relating NLR and PLR with treatment response(continuous)

| **Author** | **Year** | **Design** | **Cancer** | **Patient** | **ORR** | **DCR** | **PD** | **Comments** |
| --- | --- | --- | --- | --- | --- | --- | --- | --- |
| Guida | 2022 | RC | MELANOMA | 272 |  | Baseline, after C1  ΔNLR associated with  lower response  OR=2.779 P<0.001  ΔPLR assciated with  lower response  OR=2.022 p<0.009 | no |  |
| Guven | 2022 | RC | PAN  CANCER | 231 | Baseline, 4^th^ week post rx  NLR-L, <10% NLR increase  ORR=42.3%  NLR-H or 10% NLR increase  ORR=29.4%  NLR-H and 10% NLR increase  ORR=14.3% |  |  |  |
| Huang | 2020 | RC | NSCLC | 61 |  | After C4  NLR (L vs H)  88.9% vs 11.1% | After C4  NLR (L vs H)  73% VS 27% | P=0.296 |
| Hung | 2021 | RC | HCC | 45 |  | DCR vs PD (means)  NLR  2.9 vs 4.4 p=0.028  PLR  123.7 vs 190.7 p=0.185 |  | Mean Serum NLR and PLR higher in  PD group |

Supplementary Table 3; Summary of studies relating NLR and PLR with treatment response(continuous)

| **Author** | **Year** | **Design** | **Cancer** | **Patient** | **ORR** | **DCR** | **PD** | **Comments** |
| --- | --- | --- | --- | --- | --- | --- | --- | --- |
| Jiang | 2020 | RC | NSCLC | 76 |  | Baseline, after 5^th^ dose  NLR-N/A  PLR (L vs H)  PR/SD rate; 68.7% vs 37.0%  PLR-L; had superior rate p-0.011  #No significant difference btn  PDL1 status  #PLR better predictive marker  than PDL1 |  |  |
| Jung | 2017 | RC | MELANOMA | 104 |  | DCR vs PD (means & IQR)  NLR; 1.90 vs 2.63 p=0.003 |  | Significant higher NLR in PD than in  DCR |
| Khunger | 2018 | RC | NSCLC | 109 |  | After 2^nd^ dose  Mean NLR lower in responders  (DCR) than non-responders (PD)  P=0.037 |  |  |
| Kim | 2022 | RC | GASTRIC | 45 | NLR (L vs H)  30% vs 7.1% | NLR (L vs H)  60.0% VS 35.7% |  |  |

Supplementary Table 3; Summary of studies relating NLR and PLR with treatment response(continuous)

| **Author** | **Year** | **Design** | **Cancer** | **Patient** | **ORR** | **DCR** | **PD** | **Comments** |
| --- | --- | --- | --- | --- | --- | --- | --- | --- |
| Lee | 2021 | RC | HNSCC | 125 |  | NLR-H associated with poor response  OR=0.3 95%CI 0.11-0.84  P=0.022 |  | Multivariate analysis |
| Moller | 2022 | RC | NSCLC | 90 |  | After 3^rd^ cycle  Median NLR lower in DCR than  PD (p=0.006) |  |  |
| Mountzios | 2021 | RC | NSCLC | 672 | Cohort A-ICI monotherapy  Cohort B-ICI+CT  NLR-L significantly associated  with ALI status in Cohort A  P=0.001 | NLR-L significantly associated  with ALI status in Cohort A  P<0.001 |  |  |
| Musaelyan | 2022 | RC | -NSCLC  -MELANOMA | 45  29 |  | Baseline, 2/12 after treatment  No significant difference between responders and non-responders  **NLR**  -Baseline p=0.546  -after 2 months of treatment  p=0.132  **PLR**  -Baseline p=0.244  -After 2 months of treatment p=0.428 |  |  |

Supplementary Table 3; Summary of studies relating NLR and PLR with treatment response(continuous)

| **Author** | **Year** | **Design** | **Cancer** | **Patient** | **ORR** | **DCR** | **PD** | **Comments** |
| --- | --- | --- | --- | --- | --- | --- | --- | --- |
| Nakazawa | 2022 | RC | GASTRIC | 58 |  | Pre-rx & post rx  DCR vs PD  NLR significantly higher in PD  Pre-rx; 318 vs 4.85 p=0.045  Post-rx; 2.97 vs 5.43 p=0.025 |  |  |
| Namikawa | 2020 | RC | GASTRIC | 29 |  | Baseline & wk 2, 4, 6, 8 post rx  NLR had significant association  in wk 4, p=0.044 |  |  |
| Nenclares | 2021 | PROSPE  CIVE | HNSCC | 100 |  | NLR significantly lower in responders  P<0.001 |  |  |
| Newman | 2021 | RC | NSCLC | 137 |  | NLR (L vs H)  68.9% Vs 38.3% | NLR (L vs H)  31.1% vs 61.7% |  |
| Ohashi | 2020 | RC | MELANOMA | 16 | Baseline & wk 3, wk 6 post rx  NLR  No significant difference btn  ORR and PD  p=0.269 |  |  |  |
| Ohba | 2019 | RC | NSCLC | 32 | NLR (L vs H)  63.2% vs 15.4% | NLR (L vs H)  84.2% vs 61.5% |  |  |

Supplementary Table 3; Summary of studies relating NLR and PLR with treatment response(continuous)

| **Author** | **Year** | **Design** | **Cancer** | **Patient** | **ORR** | **DCR** | **PD** | **Comments** |
| --- | --- | --- | --- | --- | --- | --- | --- | --- |
| Park | 2021 | RC | NSCLC | 83 |  | Responders (DCB) vs non-responders (NDB)  Pre-rx;  NLR, PLR no significance  C2-C4;  NLR-L, PLR-L significant association in responders, p<0.05 |  |  |
| Petrova | 2020 | RC | NSCLC | 119 |  | NLR (L vs H)  66.2% vs 33.8% p<0.001  PLR (L vs H)  63.5% vs 36.5% p<0.001 | NLR (L vs H)  18.2% vs 81.8% p<0.001  PLR (L vs H)  27.3% vs 72.7% p<0.001 |  |
| Pu | 2021 | RC | NSCLC | 184 | NLR (L vs H)  40.9% vs 26.1% p=0.042  PLR (L vs H)  41.4% vs 28.2% p=0.062 | NLR (L vs H)  74.8% vs 56.5% p=0.010  PLR (L vs H)  79.8% vs 54.1% p=0.000 |  |  |
| Quaquarinii | 2022 | RC | NSCLC | 166 |  | NLR (L vs H)  87.6% vs 37.6% | NLR (L vs H)  15.87% vs 84.13% | PD vs DCR  OR=11.21 95%CI 5.22-26.09 P<0.0001 |

Supplementary Table 3; Summary of studies relating NLR and PLR with treatment response(continuous)

| **Author** | **Year** | **Design** | **Cancer** | **Patient** | **ORR** | **DCR** | **PD** | **Comments** |
| --- | --- | --- | --- | --- | --- | --- | --- | --- |
| Rebuzzi | 2022 | RC | RCC | 422 | Compared mean values  Baseline  NLR=3.18 PLR=184  Longitudinal variation(after 4 doses)  NLR=3.24 PLR=N/A |  | Baseline  NLR=4.12 PLR=237  After 4 doses;  NLR=5.41 PLR=N/A | Baseline  NLR p-value=0.012  PLR p-value=0.003  Significant association  After 4 doses;  NLR p-value=0.0053 (significant)  PLR p-value=0.092 (no significance) |
| Russo | 2018 | RC | NSCLC | 62 | PLR (L vs H)  46.15% vs 8.3% p<0.0004 |  |  | Significant for neutrophilia & thrombocytosis |
| Simonaggio | 2020 |  | RCC | 86 |  | NLR (L vs H) at wk 6  81% vs 40% p=0.0007 |  | Baseline NLR (DCR vs PD)  2.44 vs 3.74 p=0.048 (significant) |
|  |  |  | NSCLC | 85 | N/A | N/A | N/A | N/A |

Supplementary Table 3; Summary of studies relating NLR and PLR with treatment response(continuous)

| **Author** | **Year** | **Design** | **Cancer** | **Patient** | **ORR** | **DCR** | **PD** | **Comments** |
| --- | --- | --- | --- | --- | --- | --- | --- | --- |
| Spassova | 2021 | RC | MERKEL | 114 |  | NLR (L vs H)  49% vs 27% | NLR (L vs H)  45% vs 38% | No association done |
| Tanaka | 2022 | RC | HCC | 28 |  |  |  | Responders(OR) vs non-responders  (non-OR)  **Median NLR**  OR (CR+PR); 3.00 (1.19,6.52)  Non-OR(SD+PD); 3.10 (1.41,25.7)  p=0.214 (not significant) |
| Wang | 2022 | RC | ESCC | 69 | NLR (L vs H)  **Baseline**  36.1% s 9.1% p=0.018  **At V1**  34.4% vs 15.6% p=0.083  **Variation (baseline-V1)**  <20% vs >20%;31.6% vs 22.2%  P=0.430 | NLR (L vs H)  **Baseline**  52.8% vs39.4% p=0.265  **At V1**  59.4% vs 40.6% p=0.134  **Variation (baseline-V1)**  <20% vs >20%;52.6% vs 48.9%  P=0.784 |  |  |

Supplementary Table 3; Summary of studies relating NLR and PLR with treatment response(continuous)

| **Author** | **Year** | **Design** | **Cancer** | **Patient** | **ORR** | **DCR** | **PD** | **Comments** |
| --- | --- | --- | --- | --- | --- | --- | --- | --- |
| Wu | 2021 | RC | NSCLC | 136 |  |  |  | Univariate analysis  NLR p=0.446 PLR p=0.978  No significant relationship with  short term efficacy and immune response |
| Yamamoto | 2020 | RC | UC | 121 | NLR p=0.0160 (significant)  PLR p=0.0536 (marginal significance) |  |  |  |
| Yuequan | 2021 | RC | NSCLC | 103 |  | NLR (L vs H)  95.6% VS 82.3% p=0.042  PLR=N/A | NLR (L vs H)  33.3% vs 66.7% p=0.042  PLR=N/A | PR vs PD & SD vs PD  NLR p=0.759  PLR p=0.673  No significant relationship |

**Abbreviations**; RC-Retrospective cohort; PC-Prospective cohort; GI-Gastroinstestinal cancer; HCC-Hepatocellular carcinoma; HNSCC-Head and neck squamous cell carcinoma; RCC-Renal cell carcinoma; UC-Urothelial carcinoma; NLR-Neutrophil-to-lymphocyte ratio; NSCLC-Non-small cell lung cancer; CRC-Colorectal cancer; ESCC-Esophageal squamous cell carcinoma; PAN CANCER-Multiple cancers; PLR-Platelet-to-lymphocyte ratio; Pre-rx-Pre-treatment(baseline); Post-rx-Post-treatment; L vs H-low ratio vs high ratio; N/A-not applicable

DCB-Derived clinical benefit; NDB-No derived benefit; ICI-immuno-checkpoint inhibitors; ALI-Advanced lung cancer inflammation index ORR-Objective response rate DCR-Disease control rate; PD-Progressive disease
